# Coexistence of PFAPA syndrome and FMF in Armenian children

**DOI:** 10.1186/1546-0096-13-S1-P102

**Published:** 2015-09-28

**Authors:** N Mkrtchyan, G Amaryan, T Sarkisian

**Affiliations:** 1 National Paediatric Center for Familial Mediterranean Fever; “Arabkir” Medical Center - Institute of Child and Adolescent Health; Yerevan State Medical University, Yerevan, Armenia; 2 Centre of Medical Genetics and Primary Health Care; Yerevan State Medical University, Yerevan, Armenia

## Introduction

Familial Mediterranean Fever (FMF) is an ethnic disease for Armenians. Recurrent attacks of fever with tonsillitis are also clinical symptom of FMF, especially in early childhood. They are also characteristic of the PFAPA syndrome (periodic fever, aphthous stomatitis, pharyngitis, adenopathy), which can be confused with FMF.

## Objective

To investigate the peculiarities of the coexistence of PFAPA syndrome and FMF in Armenian children.

## Methods

28 children with recurrent episodes of fever and tonsillitis was observed at the National Pediatric Centre for FMF (19 boys, 9 girls, aged from 1 to 8 year). The diagnosis of FMF was confirmed by Tel-Hashomer criteria and molecular genetic detection of MEFV mutations. PFAPA syndrome was diagnosed based on recognized clinical criteria and excluding other causes of tonsillitis.

## Results

Coexistence of FMF and PFAPA was in 10 out of 28 patients (36%). They had earlier FMF onset (mean age of 1y. 3 mo.), frequent febrile attacks of abdominal pain and/or pleuritis, pericarditis (7 out of 10 patients), aphthous stomatitis (6 patients), tonsillitis with cervical lymphadenitis (9 children), as well as marked increase of acute inflammatory markers. An average age of PFAPA manifestation was 2 years 2 mo. MEFV gene M694V mutation was found in 8 patients out of 10 with the following distribution of genotypes:: one homozygous genotype (V726A/V726A), 5 compound heterozygotes: (M694V/V726A (2), M694V/F479L (2), M694V/E148Q (1), 4 heterozygotes (M694V/N (3 children), V726A/N (1).

Isolated PFAPA syndrome was diagnosed in 18 out of 29 children (mean age of onset - 2 years 4 mo.). They had repeated attacks of fever and tonsillitis, rare - pleuritis or pericarditis (4 children), occasionally - mild abdominal pain and a moderately increased acute phase reactants. 13 out of 18 patients were carriers of one mutation, mostly M694V/N (9 out of 13). The frequency of PFAPA attacks in both groups of patients was similar (1-2 times a month).

## Conclusion

The coexistence of PFAPA syndrome and FMF was noticed in 36% of investigated children. They were characterized by earlier FMF onset, severe course of disease with frequent febrile attacks and the prevalence of MEFV compound-heterozygous genotype. In contrast, among patients with isolated PFAPA syndrome prevailed heterozygous genotype. M694V mutation was the most frequent.

MEFV mutation genetic screening is recommended for Armenian children, especially under the age of 5, with recurrent fever and tonsillitis for early diagnosis of FMF, differential diagnosis with PFAPA syndrome, treatment and prevention of complications.

